# Spatio-temporal and trade export risk analysis of bluetongue disease in France: A case study of China

**DOI:** 10.3389/fvets.2022.955366

**Published:** 2022-11-03

**Authors:** Qiao-ling Yang, Shu-wen Zhang, Song-yin Qiu, Qiang Zhang, Qin Chen, Bing Niu

**Affiliations:** ^1^School of Environmental and Chemical Engineering, Shanghai University, Shanghai, China; ^2^School of Life Sciences, Shanghai University, Shanghai, China; ^3^Chinese Academy of Inspection and Quarantine, Beijing, China; ^4^Technology Center of Animal, Plant and Food Inspection and Quarantine, Shanghai, China

**Keywords:** France, bluetongue disease, spatio-temporal analysis, semi-quantitative risk analysis, live cattle, bovine semen

## Abstract

Bluetongue disease (BT) is a viral disease that can be introduced through imported animals and animal products, affecting local animal husbandry. In this study, the spatial and temporal patterns of BT outbreaks (outbreak: a BT infection in cattle, sheep, or goats on a farm, involving at least one infected animal) in France were analyzed and the risk of introducing bluetongue virus (BTV) into countries through trade was assessed. A spatiotemporal analysis of BT reported during the study period (2015–2018) showed that there were clustered outbreaks of BT in France in 2016 and 2017, with outbreaks concentrated from August to December. The outbreak moved eastward from the center of mainland France to surrounding countries. A semi-quantitative risk analysis framework was established by combining the likelihood assessment and consequence analysis of introducing BTV into trading countries through trade. Exemplified by China, the research showed that in the analysis of the likelihood of BTV from France being introduced into trading countries through live cattle trade, China imports a large number of live cattle, bringing high risks. The likelihood of introducing bovine semen into trading countries was similar to that of live cattle, but the harm caused by the trade in live cattle was higher than that caused by the trade in bovine semen. This risk analysis framework can provide a reference for other countries to quickly assess the risk of bluetongue transmission in import and export trade.

## Introduction

Bluetongue disease (BT) is an arthropod-borne viral disease that mainly infects ruminants (1, 2). The global distribution of the bluetongue virus (BTV) has changed since 2000, especially in Europe and the Mediterranean basin (3). In 2006, the BTV serotype 8 (BTV-8) emerged and spread in northern Europe. France was one of the first countries to be infected (4). In January 2018, France announced a transition from a BTV-free status in 2000 to an endemic status of serotypes 4 and 8 (5), which means that BTV will exist in France for a long time.

The outbreak of BT will have serious economic consequences (2). It is estimated that BT outbreaks cost the economy more than $3 billion annually (6). BT has been added to the list of notifiable infectious diseases of the Office of International Epidemiology (OIE) (7). BT can be transmitted by ruminants and their genetic material, while infected bulls can secrete BTV in their semen, and recipient cows can become infected when they receive infectious semen (8). France is one of the world's leading exporters of live cattle. According to the UN Comtrade Database, France exported a total of 1.62 million live cattle in 2020, ranking first in the world. In 2020, the trade volume of bovine animal products (bovine semen) in France reached 16,000 kg (9). Therefore, it is important to assess the risk of BTV entering importing countries through trade in animals and animal products. According to records, the BTV was first isolated in China in 1979, when a bluetongue-like disease broke out on sheep farms in Shizong County and the surrounding area in the Qujing region of Yunnan Province, and then broke out in many provinces in China (10, 11). As a major trading country, China imports BT-susceptible animals such as live cattle. It is likely to cause BTV to enter China along the trade route, thereby causing an outbreak of BT.

To assess the risk of BTV transmission through trade in animals and animal products, several studies have been conducted to analyze the epidemiological risk of BT (12, 13). Before 1990, risk assessments were usually qualitative assessments. However, the application of import and export risk analysis gradually increased since the Agreement of Sanitary and Phytosanitary Measures (“SPS Agreement”) formally established risk analysis as a tool in the selection of international trade measures (14). For example, researchers have quantitatively assessed the likelihood of BT entering the United States (15) and EU member states (16) through animal trade, as well as the transmission of BTV through bovine semen (8). However, most risk analysis experiments only evaluate one or two steps in the risk assessment framework due to the difficulty of data collection and other reasons.

In order to consider multiple aspects of the risk assessment framework steps and avoid complex data collection, this study put forward the hypothesis that there are live cattle trade between China and France. The risk of BT transmission through the trade of live cattle and bovine semen from France was analyzed. A semi-quantitative analysis combining qualitative and quantitative analyses was applied to evaluate the consequences of each potential influencing factor and disease invasion in the transport route of animals and animal products (17). At the same time, the cluster evolution of BT and the temporal and spatial trend of BT transmission in France were analyzed. The aim of this study is to provide rapid risk assessment for the animal trade and targeted animal disease prevention and control information for risk managers.

## Materials and methods

### Data preparation

The information on the time, location, and the number of affected animals of BT outbreaks were obtained from the Emergency Prevention System (EMPRES) for Transboundary Animal and Plant Pests and Diseases (http://empres-i.fao.org/eipws3g/#h=0). In this study, an outbreak was defined as a bluetongue infection in cattle, sheep, or goats on a farm, involving at least one infected animal.

### Global Moran's I

Global Moran's I (18, 19) analyzes spatial autocorrelation based on feature locations and feature values simultaneously. The spatial distribution mode of the outbreak is obtained as cluster mode, scattered mode, or random mode. The significance of the index is assessed by calculating Moran's I index as well as the z-score and *p*-value. Moran's I range from −1 (similar to chessboard distribution) to +1 (clustering distribution of similar values), 0 indicating no spatial autocorrelation. The larger the absolute value of Moran's I, the stronger the spatial autocorrelation. The global Moran index (Moran's I) is calculated as follows:


(1) $$\begin{array}{l} \text{I}=\frac{\text{N}}{{\text{S}}_{\text{O}}}\underset{\text{i}}{\sum }\underset{\text{j}}{\sum }{\text{ω}}_{\text{ij}}\frac{\left({\text{x}}_{\text{i}}-\text{u}\right)\left({\text{x}}_{\text{j}}-\text{u}\right)}{\sum_{\text{i}}{\left({\text{x}}_{\text{i}}-\text{u}\right)}^{2}} \end{array}$$

(2) $$\begin{array}{l} \text{v}{\text{S}}_{\text{O}}=\underset{\text{i}}{\sum }\underset{\text{j}}{\sum }{\text{ω}}_{\text{ij}} \end{array}$$

where N is the number of observation regions, ω_*ij*_ is the spatial weight matrix corresponding to the spatial element, *i* and *j, x*_*i*_ and *x*_*j*_ are the observations in regions *i* and *j*, and *u* is the mean value. The z-score is calculated as follows:


(3) $$\begin{array}{l} \text{z}=\frac{\text{I}-\text{E}\left[\text{I}\right]}{\sqrt{\text{V}\left[\text{I}\right]}} \end{array}$$

where $E\left[I\right]=\frac{-1}{N-1}$, V [I] = E [I^2^] − E[I]^2^.

### Getis-Ord Gi^*^

Getis-Ord Gi^*^ is used to evaluate each feature in the context of adjacent features and then compare the local situation with the global situation (20). When a feature has a high value and is surrounded by other high-value features, and the GI ^*^ value is positive and significant, it is a statistically significant hot spot. On the contrary, when the GI ^*^ value is negative and significant, it indicates a cold spot. The formula for calculating the $G_{i}^{*}$ index is as follows:


(4) $$\begin{array}{l} G_{i}^{*}=\frac{\sum_{j=1}^{n}w_{ij}x_{j}-\sum_{j=1}^{n}w_{ij}\bar{x}}{S\sqrt{\frac{\left(n\sum_{j=1}^{n}x_{ij}^{2}-{\left(\sum_{j=1}^{n}w_{ij}\right)}^{2}\right)}{n-1}}} \end{array}$$

where *w*_*ij*_ is the spatial weight between elements *i* and *j, xj* is the density of element *j*, and n is the number of data points. $\bar{x}=\frac{\sum_{j=1}^{n}xj}{n}$, $S=\sqrt{\frac{\sum_{j=1}^{n}x_{j}^{2}}{n}}{\left(\bar{x}\;\right)}^{2}$.

### Spatiotemporal scan analysis

In order to study the relationship between time and space in the French BT epidemic, this study adopts the spatiotemporal scanning analysis method (21, 22) to analyze the BT epidemic in France from 2015 to 2018. In the identification of spatiotemporal clusters, the scanning window is set to scan in the spatiotemporal scope to find statistically significant high outbreak clusters. By employing a moving window, a dynamic cylindrical window is established within the study area (the bottom of the cylinder represents the geographic area and the height represents the length of time that the outbreak may last). The log-likelihood ratio (LLR) and relative risk (RR) of each scanned cylinder are calculated by discrete Poisson models, representing the relationship between the risk of injury occurring within the cluster and the risk outside (23). When it is found that the probability of an outbreak inside the cylinder is higher than that outside the cylinder, it can be considered that the scanning cylinder is a spatiotemporal cluster, that is, there is a potential outbreak. The spatiotemporal scanning uses SaTScan 9.4.2 software to scan statistics. The information about the outbreak of BT in France was imported into the input window, including the number of outbreaks, the year and month of the outbreak, and the number of cattle and sheep in each province. In the analysis window, the analysis type was selected as space-time, and the probability model was selected as space-time displacement. In the advanced settings, the maximum spatial cluster size was set to 1,000 km, and the number of Monte Carlo sampling iterations was set to 999. The maximum scan window of the spatial scan statistic was set as a proportion of the total population at risk, and different percentages were selected for calculation, such as 50, 40, 30, 20, and 10%. In the output window, the output path and output file format (selected ShapeFile) were specified. Finally, ArcMap 10.1 was applied to visualize the results.

### Standard deviation ellipse analysis

Standard deviational ellipse (SDE) analysis (24) is used to reveal the distribution characteristics of features in space. The ellipse spatial distribution range represents the main area of the spatial distribution of geographic elements, the center represents the relative position of disease distribution, the azimuth angle reflects the main trend direction of disease distribution, and the long and short axes represent the degree of deviation from the center in the direction. Depending on whether the ellipse is elongated, the direction of the outbreak is analyzed. The formula for calculating the ellipse is as follows:


(5) $$\begin{array}{l} \text{SD}{\text{E}}_{\text{a}}=\sqrt{\frac{\sum_{\text{i}=1}^{\text{n}}{\left({\text{a}}_{\text{i}}-\bar{\text{A}}\right)}^{2}}{\text{n}}} \end{array}$$

(6) $$\begin{array}{l} \text{SD}{\text{E}}_{\text{b}}=\sqrt{\frac{\sum_{\text{i}=1}^{\text{n}}{\left({\text{b}}_{\text{i}}-\bar{\text{B}}\right)}^{2}}{\text{n}}} \end{array}$$

where *a*_*i*_ and *b*_*i*_ are the spatial position coordinates of each element, $\bar{A}$ and $\bar{B}$ represent the arithmetic mean center, and then the direction of the ellipse is determined. Taking the X-axis as the standard, the north direction is 0°, and the calculation formula for the clockwise rotation is as follows:


(7) $$\begin{array}{l} \tan\theta =\frac{\sqrt{({\left(\sum_{i=1}^{n}\alpha_{i}{\;}^{2}-\sum_{i=1}^{n}\beta_{i}{\;}^{2}\right)}^{2}+\left(\sqrt{\left({\left(\sum_{i=1}^{n}\alpha_{i}{\;}^{2}-\sum_{i=1}^{n}\beta_{i}{\;}^{2}\right)}^{2}+4{\left(\sum_{i=1}^{n}\alpha_{i}\beta_{i}\right)}^{2}\right)}\right)}}{2\left(\sum_{i}^{n}\alpha_{i}\beta_{i}\right)} \end{array}$$

where α_*i*_ and β_*i*_ are the coordinate differences between the average center and *a*_*i*_and *b*_*i*_, respectively. Finally, the standard deviation of the X and Y axis are calculated as follows:


(8) $$\begin{array}{l} {\text{σ}}_{\text{a}}=\sqrt{2}\sqrt{\frac{{\left({\text{α}}_{\text{i}}\cos\text{θ}-{\text{β}}_{\text{i}}\sin\text{θ}\right)}^{2}}{\text{n}}} \end{array}$$

(9) $$\begin{array}{l} {\text{σ}}_{\text{a}}=\sqrt{2}\sqrt{\frac{{\left({\text{α}}_{\text{i}}\cos\text{θ}-{\text{β}}_{\text{i}}\sin\text{θ}\right)}^{2}}{\text{n}}} \end{array}$$

### Linear directional mean

Linear directional mean (LDM) is centered on the average center of the centroids of all input vectors, and the length is equal to the average length of all input vectors (25). The final result has the average direction and average length of all input vectors, which are used to indicate the direction of the outbreak. The formula for calculating the linear directional mean is as follows:


(10) $$\begin{array}{l} \text{LDM}=\text{arc}\tan\frac{\sum_{\text{i}=1}^{\text{n}}\sin{\text{θ}}_{\text{i}}}{\sum_{\text{i}=1}^{\text{n}}\cos{\text{θ}}_{\text{i}}} \end{array}$$

where θ_*i*_ is the direction in which a single set of polyline elements start. All operations were done in ArcGIS 10.1.

### Semi-quantitative risk analysis

**Figure 1:**
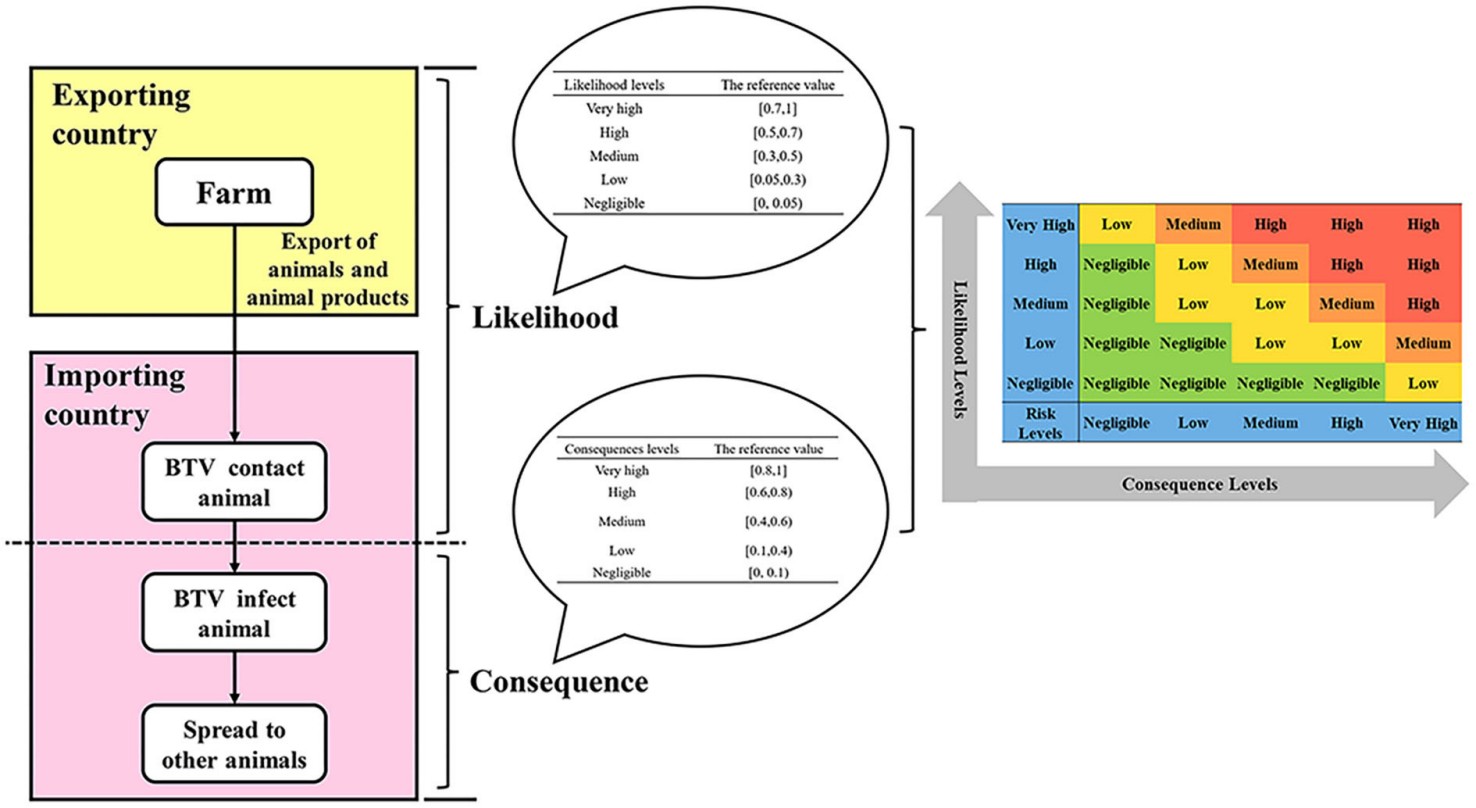
The semi-quantitative risk assessment process for imported animals and animal products into BTV.

The main idea of semi-quantitative risk analysis (26) of imported animals and animal products into BTV is to divide the overall process of importing animals and animal products, which is mainly divided into two parts: the likelihood level and the consequence level (Figure 1). Through literature analysis, brainstorming, and expert investigation, the influencing factors of the likelihood assessment and consequence of animals and animal products introduced into BTV were screened and determined, and the weight coefficients and scoring standards were established. The influencing factors of each type of parameter were described in grades, and the influential factors were scored according to the description in the evaluation and analysis process. The influencing factors and weighted scores were calculated (Table 1), and the calculated results were based on the risk event description score table to determine the likelihood and consequence of the degree of harm (Tables 2, 3). Combining the results of the two parts, the risk matrix was used to evaluate the risk level (Figure 1), and the risk level of BTV entering China through imported animals and animal products was given. The risk was divided into four levels: negligible, low-risk level, medium-risk level, and high-risk level.

**Table 1:** Score analysis of influencing factors for the semi-quantitative model.

**Parameter**	**Influencing** **factors (IFi)**	**Influencing factors** **score (Si)**	**Weight** **factor (Wi)**	**Weighted scores** **for influential** factors (Si*Wi)
Parameter 1	IF 1	S_1_	W_1_	${\text{S}}_{1}^{*}$W_1_
	IF 2	S_2_	W_2_	${\text{S}}_{2}^{*}$W_2_
	IF 3	S_3_	W_3_	${\text{S}}_{3}^{*}$W_3_
Parameter 2	IF 4	S_4_	W_4_	${\text{S}}_{4}^{*}$W_4_
	IF 5	S_5_	W_5_	${\text{S}}_{5}^{*}$W_5_
	IF 6	S_6_	W_6_	${\text{S}}_{6}^{*}$W_6_
……	……	……	……	……
Parameter x	IF n	S_n_	W_n_	${\text{S}}_{\text{n}}^{*}$W_n_
Total weighted score				PA$=\sum_{i=1}^{n}S_{i}W_{i}$
Weighted full score				PB$=\sum_{i=1}^{n}{S_{\max}W}_{i}$
Result				$P=\frac{PA}{PB}=\frac{\sum_{i=1}^{n}S_{i}W_{i}}{\sum_{i=1}^{n}{S_{\max}W}_{i}}$

**Table 2:** The assessment of likelihood levels in risk events.

**Likelihood levels**	**The reference value of the probability interval for the occurrence of the risk event**	**Definition**
Very high	[0.7, 1]	The event is almost certain to occur
High	[0.5, 0.7]	The event occurs frequently
Medium	[0.3, 0.5]	The event occurs on occasion
Low	[0.05, 0.3]	Events are rare but cannot be ruled out
Negligible	[0, 0.05]	The probability of the event occurring is small and not worth considering; if there is, it is not expected to occur for many years

**Table 3:** The assessment of consequence levels in risk events.

**Consequences levels**	**The reference value of the probability interval for the occurrence of the risk event**	**Definition**
Very high	[0.8, 1]	The consequences are very serious
High	[0.6, 0.8)	The consequences are serious
Medium	[0.4, 0.6)	It has a certain impact, and it is necessary to take preventive measures for the possible consequences
Low	[0.1, 0.4)	It is insignificant, but unavoidable
Negligible	[0, 0.1)	It doesn't matter, no need to think about it

## Results

### Statistics of BT outbreaks in France

**Figure 2:**
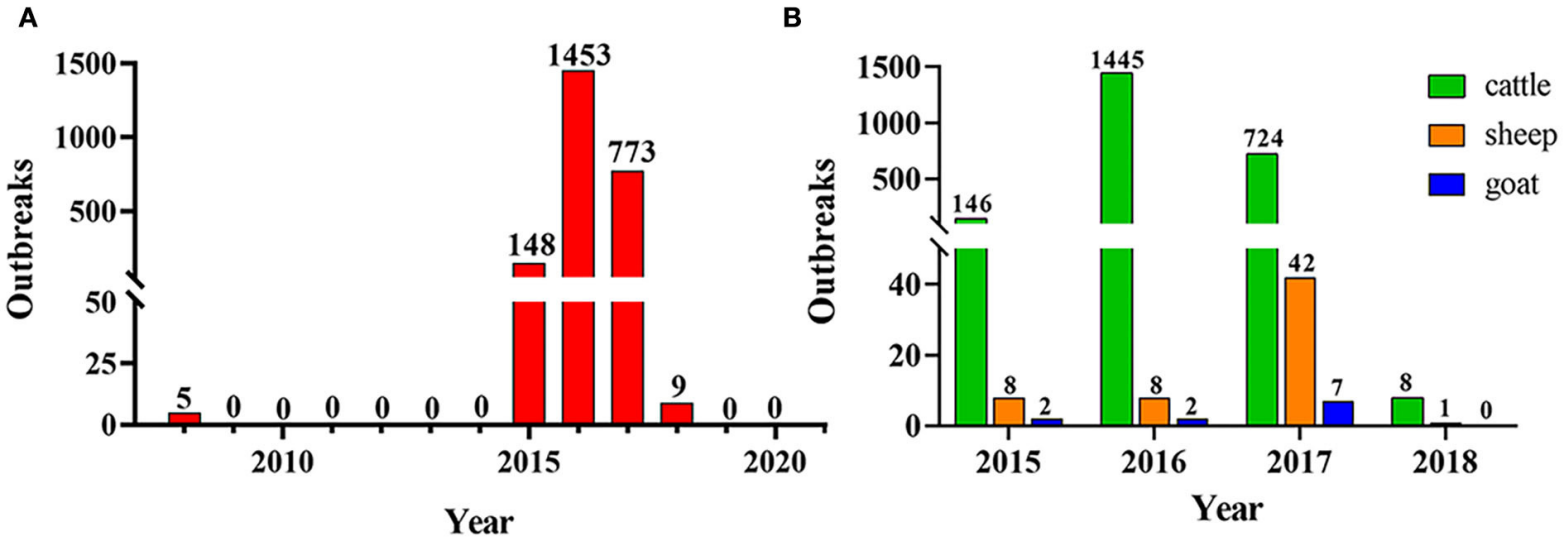
The epidemic situation of BT in France. **(A)** Outbreaks of BT in France from 2008 to 2019; **(B)** BT-infected animals in France from 2015 to 2018.

As shown in Figure 2A, there were 2,388 outbreaks of BT in France from 2008 to 2020. The first outbreak of BT occurred in France in 2008, and it has not recurred for the next 6 years. However, there was a re-outbreak of BT in France in 2015, and the outbreak peaked in 2016, after which the number of outbreaks declined. No BT outbreaks were reported in France in 2019 and 2020. During the outbreak of BT in France from 2015 to 2018, cattle were the most commonly infected animals (Figure 2B). The monthly outbreaks of BT in France from 2015 to 2018 were calculated (Table 4). The outbreak of BT in France has obvious seasonality. The peak season for BT outbreaks was from September to December. The number of outbreaks was the highest in November, and the number of outbreaks decreased significantly in June and July.

**Table 4:** Monthly outbreaks of BT in France in 2015–2018.

**Month**	**2015**	**2016**	**2017**	**2018**
Jan	0	15	79	9
Feb	0	51	113	0
Mar	0	39	140	0
Apr	0	20	58	0
May	0	13	22	0
Jun	0	1	0	0
Jul	0	5	0	0
Aug	1	13	32	0
Sep	45	126	148	0
Oct	33	306	109	0
Nov	33	499	10	0
Dec	36	367	62	0

**Figure 3:**
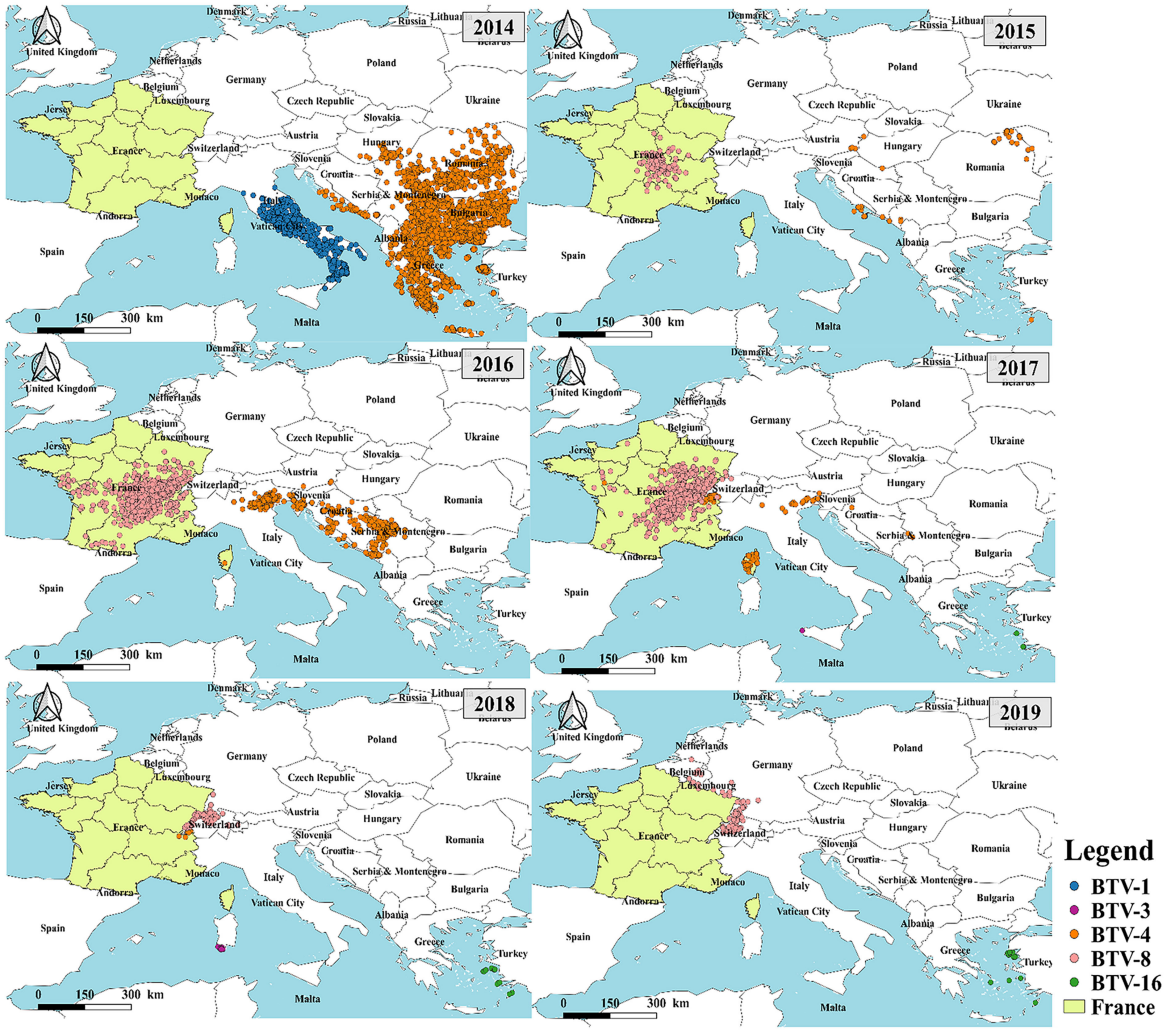
Distribution of serotypes of BT in France and surrounding countries from 2014 to 2019.

To analyze the relationship between the outbreaks of BT in France and the rest of Europe, a statistical analysis of the serotypes of BT outbreaks in Europe was performed (Figure 3). According to the data from OIE statistics, the major outbreaks of BT serotypes in Europe were BTV-1, BTV-3, BTV-4, BTV-8, and BTV-16. The results showed that the outbreak of BT in 2014 was concentrated in southern Europe including Italy (BTV-1), Greece, Bulgaria, Romania, Serbia (BTV-1 and BTV-4) except France. From 2015 to 2018, the major serotype leading to the BT outbreak in mainland France was BTV-8, which was gradually shifted to neighboring countries including Switzerland, Germany, Belgium, and other countries. The major serotype responsible for these outbreaks was BTV-8. This indicated that the outbreak of BT in mainland France and its surrounding areas was different from the BTV outbreak in southern Europe in terms of serotypes.

### Spatial-temporal distribution characteristics of BT in France

The data of the BT outbreak have two dimensions, time and space. In this study, in addition to the analysis of spatial aggregation, the change in the epidemic aggregation area and the direction of epidemic movement over time were analyzed.

#### Analysis of agglomeration

**Figure 4:**
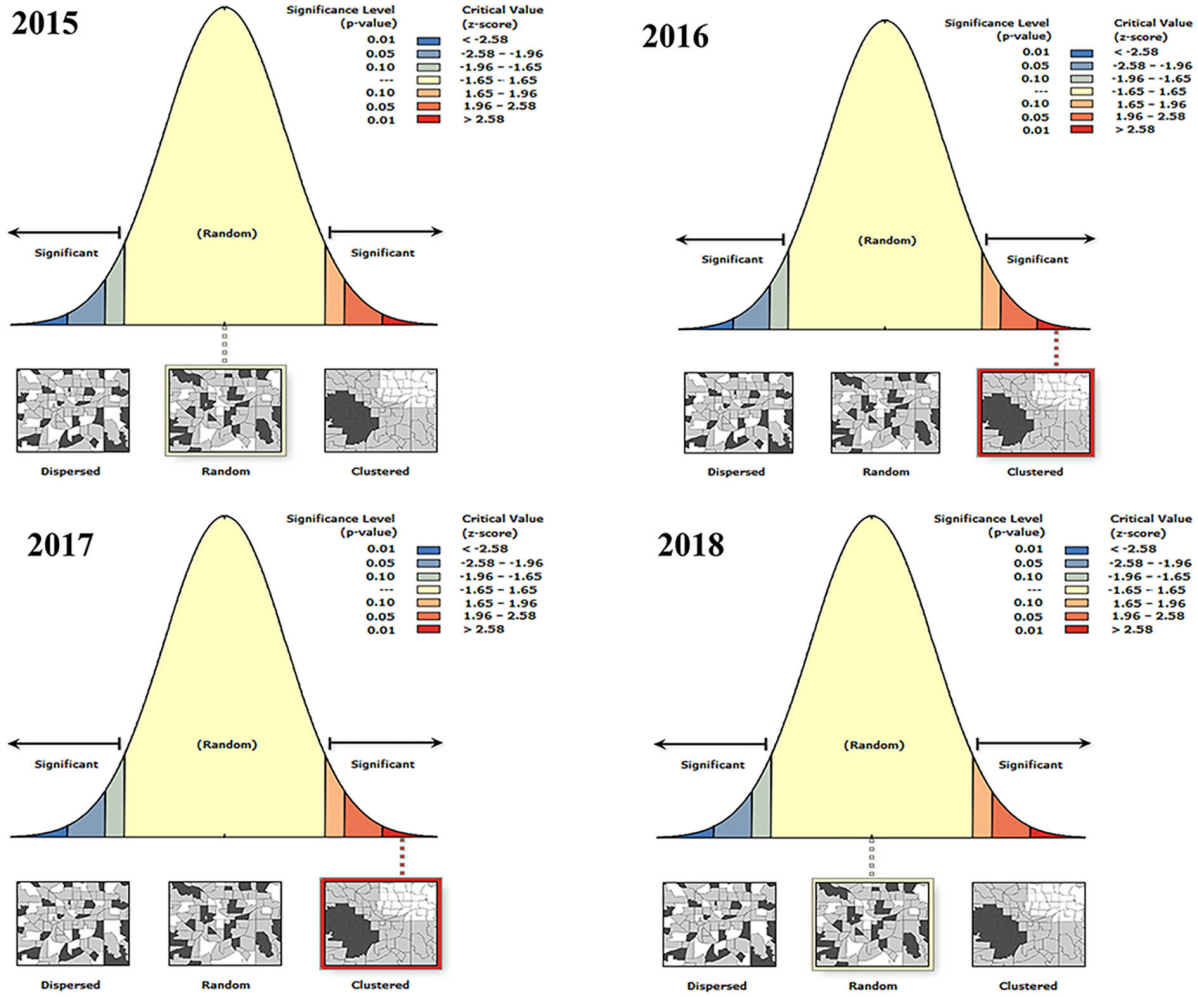
Global autocorrelation analysis of BT in France from 2015 to 2018.

The aggregation of BT outbreaks in France was analyzed by year (Figure 4 and Table 5). The spatial distribution of BT outbreaks in 2015 and 2018 showed random distribution without clustering. In 2016 and 2017, Moran's I was positive and the z-scores (multiples of the standard deviation) were 19.29 and 13.36, respectively, indicating that the similar attributes with high outbreaks around the high outbreak area were clustered together and have obvious clustering characteristics. *P* = 0 indicates that the result was not generated by random data, and the generated result was credible. In 2016 and 2017, the outbreaks of BT in France were spatially correlated and clustered.

**Table 5:** Global autocorrelation parameters for BT in France from 2015 to 2018.

	**2015**	**2016**	**2017**	**2018**
Moran's I	−0.005224	0.004111	0.019324	−0.151741
Expectation index	−0.003333	−0.000689	−0.001295	−0.125
Variance	0.000006	0.000001	0.000002	0.005096
z-score	−0.78	19.29	13.36	−0.37
*P* value	0.43	0	0	0.71
R/C	R	C	C	R

R is random distribution, C is clustered distribution.

**Figure 5:**
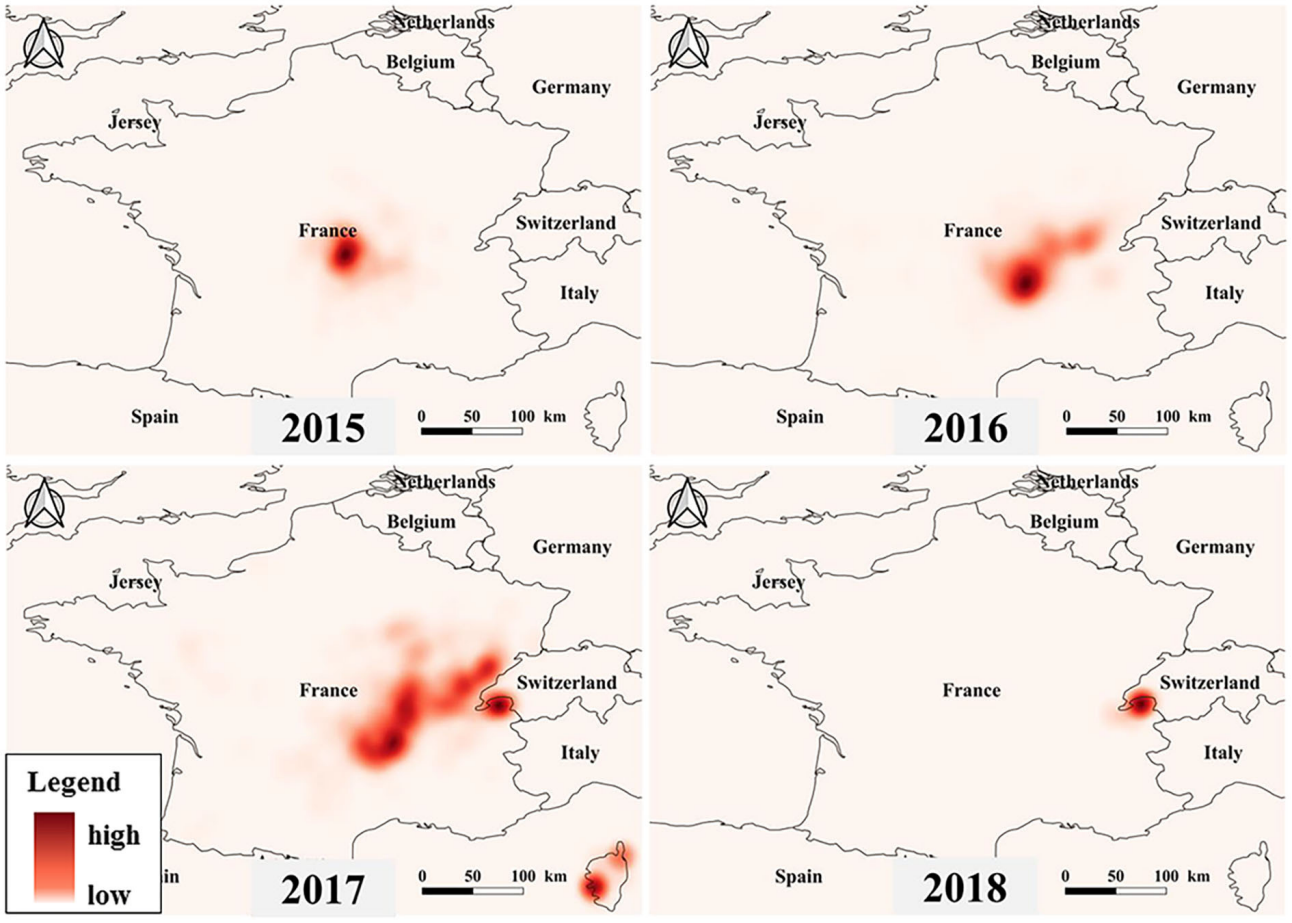
Heat map of BT outbreak in France from 2015 to 2018.

The heat map shows the severity of the outbreak of BT in France, with areas in dark colors showing serious outbreaks (Figure 5). The results indicated that since 2015, BT has broken out in central France and started to spread and it had spread to many parts of France by 2017. By 2018, the BT outbreak hotspots shifted from the central to the French border area. It is worth noting that a severe BT outbreak occurred in Corsica, France, in 2017.

**Figure 6:**
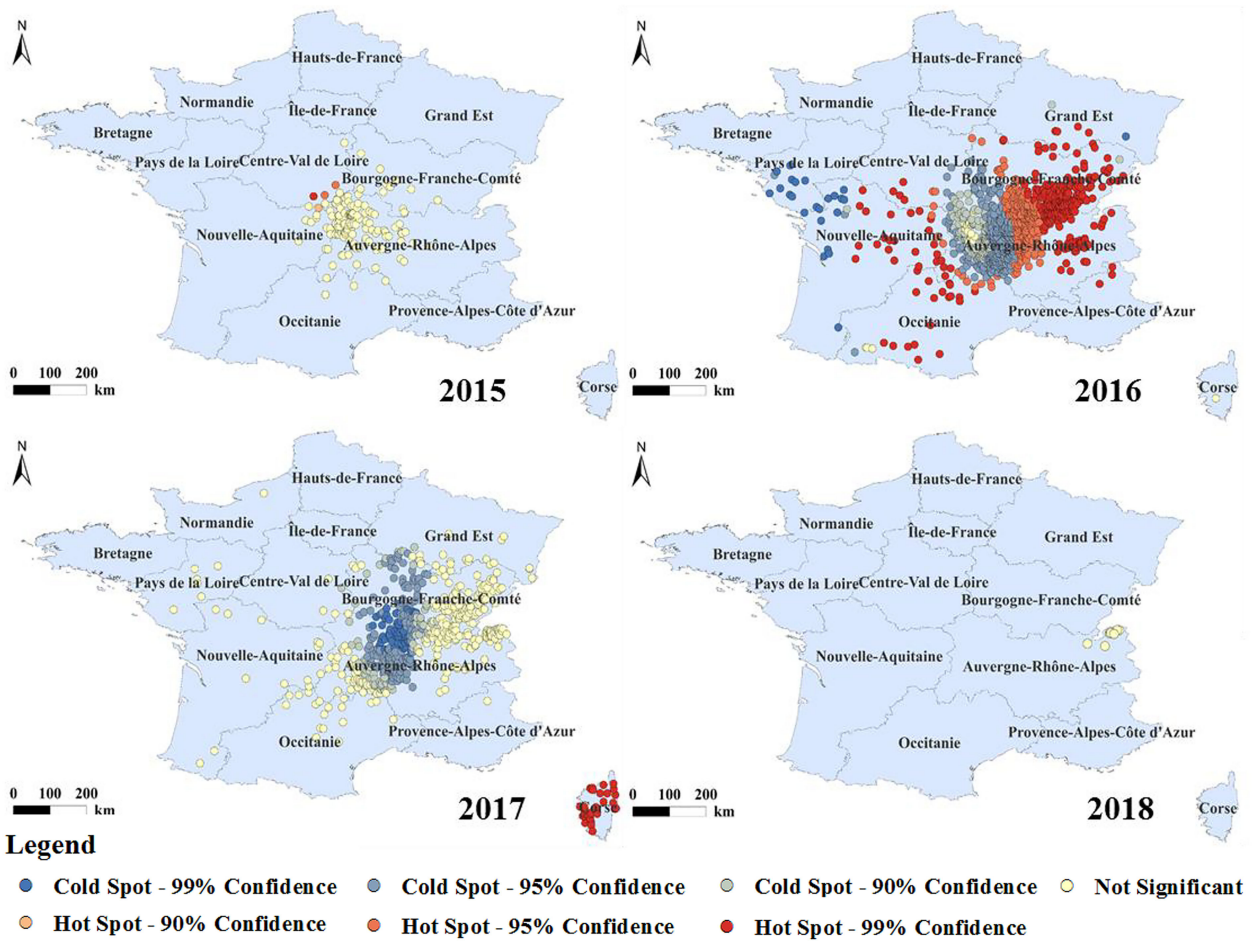
Analysis of the hot and cold spots of BT in France from 2015 to 2018.

The cold and hot spot areas of the BT outbreak with spatial significance were analyzed (Figure 6). The hot spots of BT were mainly distributed in the Midi-Pyrénées, Nouvelle-Aquitaine, Auvergne-Rhône-Alpes, Burgundy-Franche-Comté, and Corsica. According to the time of the outbreak of BT in France, outbreak of BT in France began in 2015, but the number of outbreaks was small, so the area where the outbreak started was a cold spot. In 2016, a large number of outbreak hotspots appeared in the surrounding areas, and the epidemic began to spread from the center to the surrounding areas, among which the Burgundy-Franche-Comté and Auvergne-Rhône-Alpes regions had serious outbreaks. In 2017, BT in mainland France was located in the cold spot area, but the outbreak in Corsica changed from the original cold spot to a hot spot. By 2018, the outbreaks were concentrated in the eastern part of mainland France and all were cold spots. The epidemic form of BT was constantly changing during the observation period. With the development of the epidemic, the outbreak hotspots of BT in France generally showed a trend of first rising and then falling.

**Figure 7:**
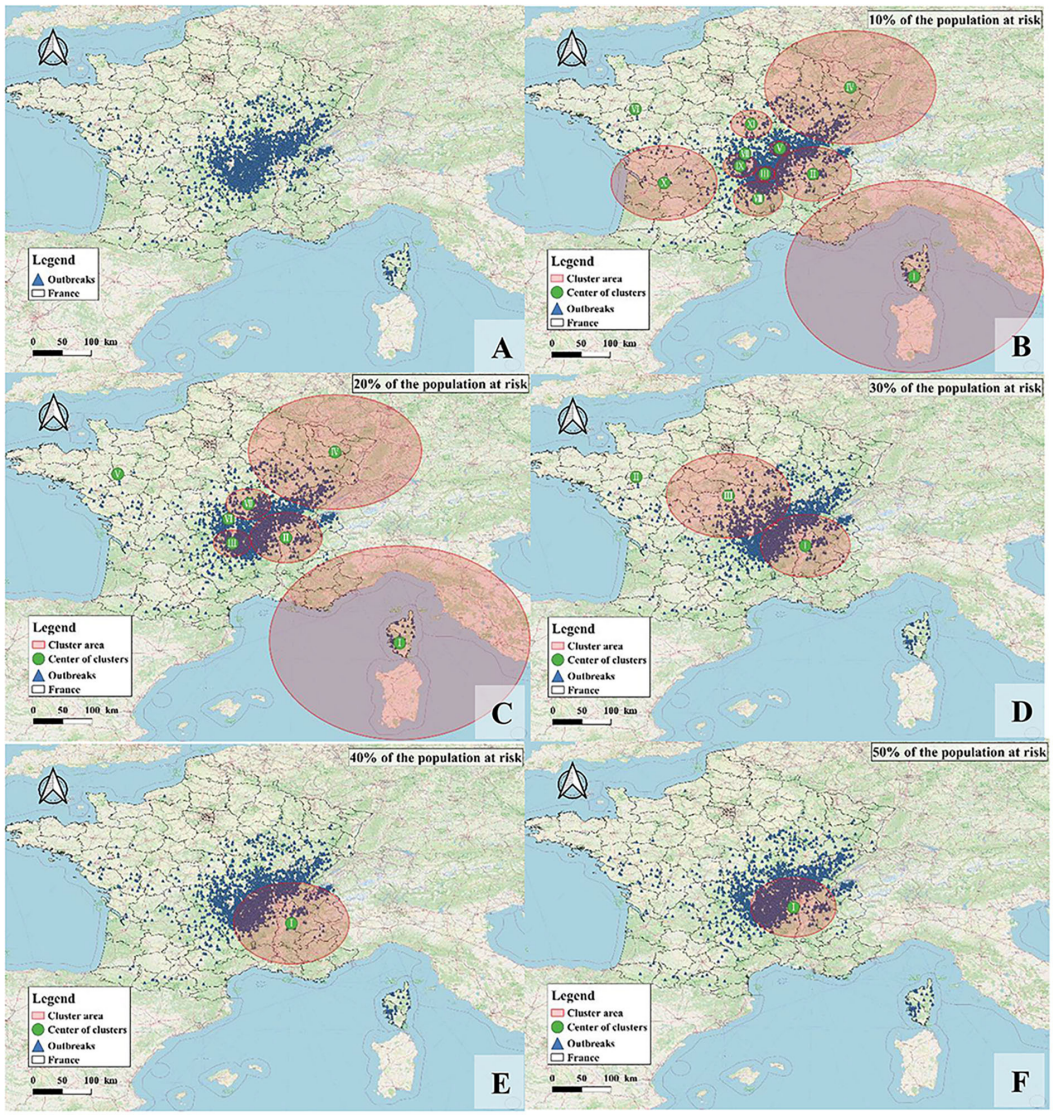
The geographical location of BT clusters in France from 2015 to 2018. **(A)** Distribution of BT in France; **(B–F)** are cluster distributions when 10–50% of animals are at risk of BT infection, respectively.

The monthly spatiotemporal scan statistics of France from 2015 to 2018 were performed to determine the high-risk areas and time of BT. Combining the spatial and temporal clustering analysis, it was found that the number of clusters decreased gradually with the increase in the percentage of animals at risk, but there were clusters in southern France (Figure 7). The obtained clusters were ordered according to the log-likelihood ratio statistic (LLR) from high to low (Table 6). Under conditions where 10 and 20% of animals are at risk, Corsica was most likely to cluster, with the most likely clustering occurring in August–September 2017. Clustering occurred in southeastern France in September–December 2016 when the percentage of animals at risk was above 40%. There was a clustering area with a radius of 0 km. Although there were only two outbreaks, the number of cases involved was high, so it had a higher relative risk (RR). Although the proportion of animals at risk was constantly changing, the Auvergne-Rhône-Alpes region in southeastern France has consistently clustered clusters, indicating a severe outbreak in this region, including Ain, Rhône, Loire, Haute-Loire, Ardèche, Drôme, and Isère.

**Table 6:** Statistical results of all clusters of spatiotemporal scanning features.

**Percentage of** **population at risk**	**Cluster ID**	**Time**	**Radius (km)**	**Observed cases**	**Expected cases**	**RR**	**LLR**
10%	I	2017/8–2017/9	377.34	242	2.43	106.87	882.09
	II	2016/10–2017/3	106.05	448	68.81	7.33	482.14
	III	2016/10–2016/12	28.93	227	34.64	6.94	239.87
	IV	2017/9–2017/10	222.91	145	13.52	11.15	215.04
	V	2016/11–2016/12	27.81	175	22.56	8.12	209.49
	VI	2017/5	0	46	0.20	238.34	205.68
	VII	2015/8–2015/9	5.17	71	1.81	40.01	192.01
	VIII	2016/10–2016/12	68.49	168	34.53	5.06	134.97
	IX	2016/11–2016/12	46.17	89	22.76	3.99	55.75
	X	2016/2	146.9	16	1.20	13.43	26.72
	XI	2016/11	54.27	16	1.71	9.39	21.51
20%	I	2017/8–2017/9	377.34	242	2.43	106.87	882.09
	II	2016/10–2016/12	97.73	573	69.43	9.69	745.14
	III	2016/10–2016/12	51.57	333	68.89	5.24	271.13
	IV	2017/9–2017/10	222.91	145	13.52	11.15	215.04
	V	2017/5	0	46	0.20	238.34	205.68
	VI	2015/8–2015/9	5.17	71	1.81	40.01	192.01
	VII	2016/11–2016/12	59.74	176	31.06	5.92	163.45
30%	I	2016/10–2016/12	123.74	797	104.20	9.64	1005.73
	II	2017/5	0	46	0.20	238.34	205.68
	III	2016/11–2016/12	166.03	245	68.90	3.75	139.35
40%	I	2016/9–2016/12	160.28	1,068	182.98	7.99	1131.02
50%	I	2016/9–2016/12	115.89	1,263	230.75	8.04	1300.67

#### Analysis of propagation direction

**Figure 8:**
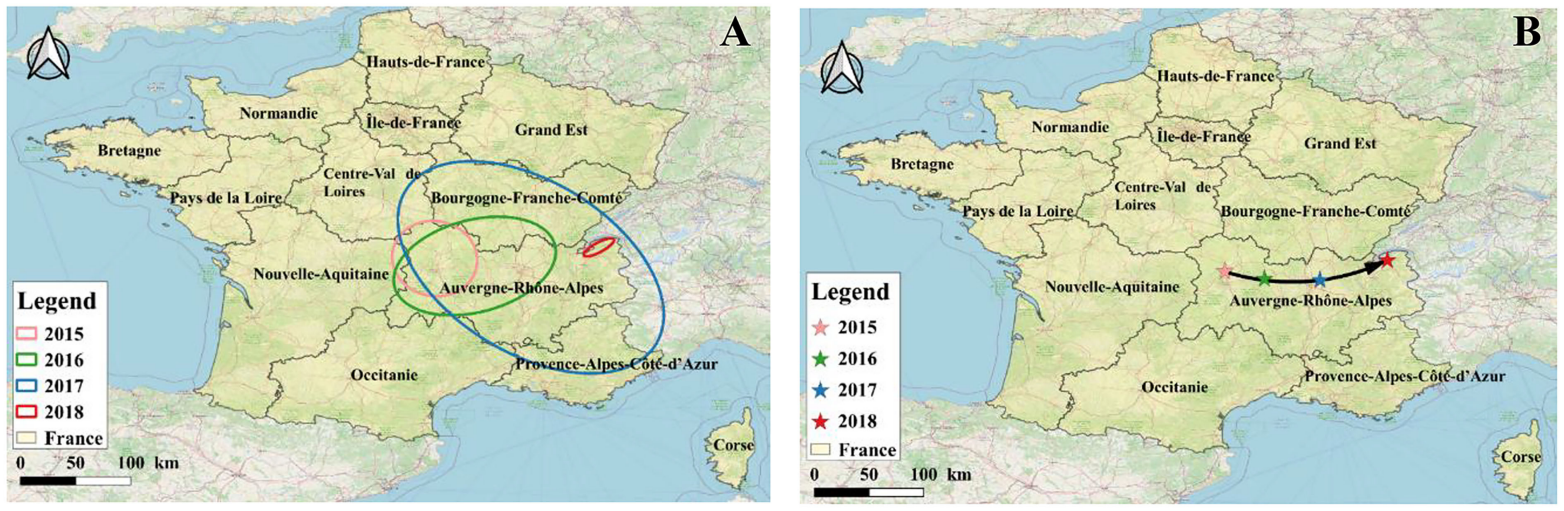
Analysis of the transmission direction of BT in France from 2015 to 2018. **(A)** SDE, standard deviational ellipse; **(B)** MC, mean center.

According to the standard deviation ellipse analysis results, the long axis of the ellipse represents the direction of data distribution, and the short axis represents the range of data distribution. The larger the oblateness, the clearer the direction of the epidemic (Figure 8). It can be seen from Figure 8 that between 2015 and 2018, the ellipse area of BT in 2017 was the largest, thus the outbreak was the most widespread and serious in France. The overall coverage of the standard deviation ellipse in 2017 was mainly located in the Burgundy-Franche-Comté and Auvergne-Rhône-Alpes regions. According to Table 7, although the area of the ellipse was the smallest in 2018 (mainly located in the Auvergne-Rhône-Alpes region), the oblateness was the largest, indicating that the outbreak has the most obvious direction in 2018. The ellipse flattening generated from 2015 to 2018 gradually increased, and the trend of the epidemic became more obvious. According to the average center analysis (Figure 8B), the spatial distribution of BT outbreaks from 2015 to 2018 showed a pattern that gradually moved eastward from the center, and gradually shifted eastward to the border areas.

**Table 7:** Related parameters of SDE and MC of BT in France from 2015 to 2018.

**Year**	**Shape area**	**CenterX**	**CenterY**	**Long axis**	**Shot axis**	Oblateness*	**Rotation angle (°)**
2015	2.06	3.088145	46.028533	0.86	0.76	0.12	100.1
2016	4.91	3.897606	45.879424	1.67	0.93	0.44	75.41
2017	16.18	5.030139	45.852749	2.97	1.73	0.42	121.66
2018	0.11	6.405672	46.256046	0.34	0.1	0.71	61.9

Oblateness* = (Long axis − Short axis)/Long axis.

### Semi-quantitative risk analysis of BTV in France introduced into China *via* live cattle

In order to analyze the risks of French trade exports to the trading countries, corresponding nodes were set up from export to import countries. Considering the large number of French trading countries, the prevalence of BT is different in trading countries. Therefore, taking China as the research object, the risk of introducing BTV in France through imported live cattle was evaluated.

#### Likelihood levels of live cattle trade introduced into BTV

Since China has had no live cattle trade with France in the past 20 years, two situations were assumed: China imports a small number of live cattle (only about 1% of China's total imported live cattle, about 2600) and a large number of live cattle (accounting for nearly 50% of China's total live cattle imports, about 1,30, 000) from France. The risk assessment of the likelihood of introducing BTV from the live cattle trade was scored and counted according to the content and scoring basis of Supplementary Table S1, and the risk assessment score of the likelihood of introducing BTV through the live cattle trade was obtained as ${\text{PA}1}_{\text{The likelihood of introducing live cattle }}=\sum_{i=1}^{n}{S_{i}W}_{i}=72$ (import a small number of live cattle) and ${\text{PA}2}_{\text{The likelihood of introducing live cattle}}=\sum_{i=1}^{n}{S_{i}W}_{i}=78$ (importing a large number of live cattle). The full weighted score for the likelihood of introduction of BTV through live cattle trade was $\text{P}{\text{B}}_{\text{The likelihood of introducing live cattle}}=\sum_{\text{i}=1}^{\text{n}}{{\text{S}}_{\max}\text{W}}_{\text{i}}=154$. Divide the obtained evaluation score by the full score, and finally obtain the likelihood of importing a small number of live cattle into BTV: $\text{P}{\text{A}}_{\text{The likelihood of introducing live cattle }}=\frac{\text{P}{\text{A}}_{\text{The likelihood of introducing live cattle}}}{\text{P}{\text{B}}_{\text{The likelihood of introducing live cattle}}}=\frac{\sum_{\text{i}=1}^{\text{n}}{\text{S}}_{\text{i}}{\text{W}}_{\text{i}}}{\sum_{\text{i}=1}^{\text{n}}{{\text{S}}_{\max}\text{W}}_{\text{i}}}=\frac{72}{154}=0.47.$ Combined with the assessment of likelihood levels in risk events (Table 2), the likelihood of risk event occurrence was medium. Assessing the likelihood of importing large quantities of live cattle into BTV: ${\text{P}}_{\text{The likelihood of introducing live cattle}}=\frac{\text{P}{\text{A}}_{\text{The likelihood of introducing live cattle}}}{\text{P}{\text{B}}_{\text{The likelihood of introducing live cattle}}}=\frac{\sum_{\text{i}=1}^{\text{n}}{\text{S}}_{\text{i}}{\text{W}}_{\text{i}}}{\sum_{\text{i}=1}^{\text{n}}{{\text{S}}_{\max}\text{W}}_{\text{i}}}=\frac{78}{154}=0.51$. According to Table 2, the likelihood level of the risk event was high.

#### Consequences levels of live cattle trade introduced into BTV

According to Supplementary Table S2, the consequences of importing live cattle from China into BTV were scored, and the score was *PA*_The consequence of introducing live cattle_ = 61. The full score of the weighted score for the analysis of the consequences of imported live cattle into BTV was $\text{P}{\text{B}}_{\text{The consequence of introducing live cattle}}=\sum_{\text{i}=1}^{\text{n}}{{\text{S}}_{\max}\text{W}}_{\text{s}}=90$. Divide the obtained score by the full score value, and finally get the consequences of live cattle trade into BTV as follow ${\text{P}}_{\text{The consequence of introducing live cattle}}=\frac{\text{P}{\text{A}}_{\text{ The consequence of introducing live cattle}}}{\text{P}{\text{B}}_{\text{The consequence of introducing live cattle}}}=\frac{\sum_{\text{i}=1}^{\text{n}}{\text{S}}_{\text{s}}{\text{W}}_{\text{s}}}{\sum_{\text{i}=1}^{\text{n}}{{\text{S}}_{\max}\text{W}}_{\text{s}}}=\frac{61}{90}=0.68$. Combined with the assessment of consequence levels in risk events (Table 3), the consequence level of the risk event was high.

#### Risk levels of live cattle trade introduced into BTV

**Figure 9:**
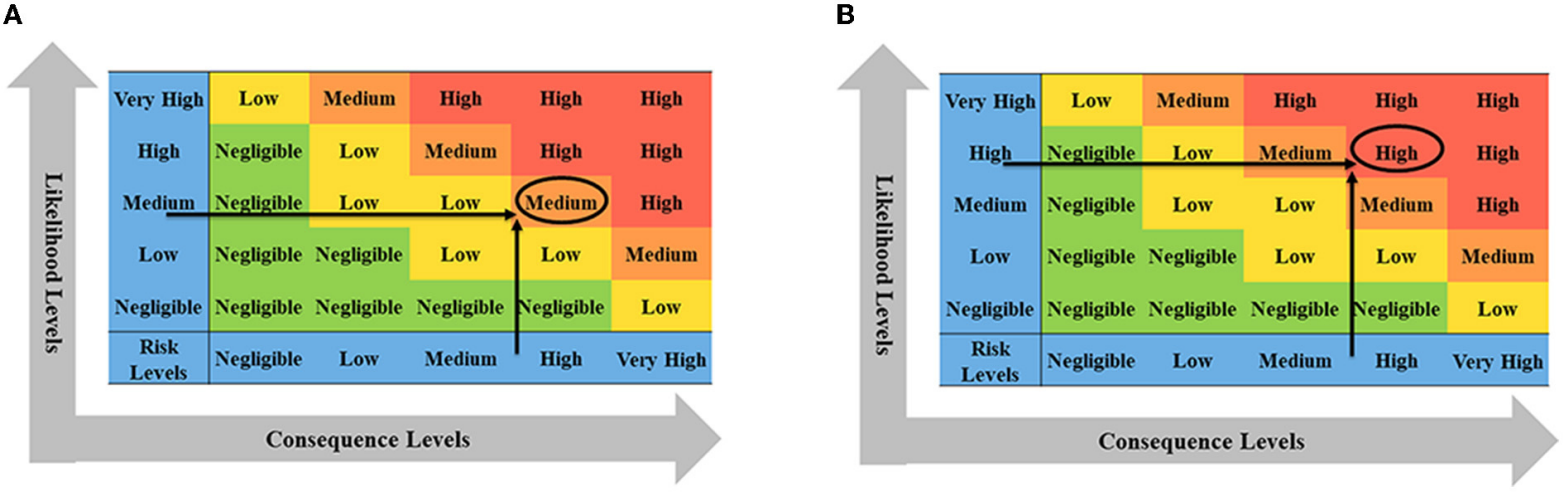
The risk determination matrix of French BTV introduced into China *via* live cattle. **(A)** China imports a small number of live cattle from France, accounting for <1% of the total imports; **(B)** China imports a large number of live cattle from France, accounting for about 99% of the total imports.

According to the transportation process of live cattle from France to China, the introduction of BTV is divided into likelihood analysis and consequence evaluation. If China only imports a small number of live cattle from France (only about 1% of China's total imported live cattle, about 2600), the likelihood level of a risk event was medium and the consequence level was high. Combining with the risk matrix in Figure 9A, it is obtained that the risk level of BTV in France entering China *via* live cattle was medium. If China imports a large number of live cattle from France (accounting for nearly 50% of China's total live cattle imports, about 130, 000), the likelihood level of risk events was high and the consequence level was high, then the risk level of BTV in France being introduced into China *via* live cattle was high (Figure 9B).

### Semi-quantitative risk analysis of introducing BTV in France into China *via* bovine semen

Bovine semen is another animal product that may spread BT. The process of importing bovine semen from France to China was analyzed to evaluate the risk of BTV being introduced into China through bovine semen. Similar to Section Semi-quantitative risk analysis of BTV in France introduced into China *via* live cattle, we divide the risk assessment into two parts: likelihood level and consequence level assessment, and finally combine the two parts to obtain the risk of BTV in France into China *via* bovine semen.

#### Likelihood levels of introducing BTV in bovine semen trade

France is one of the main countries for China to import bovine semen in Europe (27). According to the United Nations International Trade Statistics Database (28), from 2014 to 2018, China imported 539 kg of bovine semen from France. Semen from viremia animals is infectious, and recipient cows can become infected when they receive infectious semen (29–31). Therefore, importing semen from France carries certain risks. Analysis of the likelihood of imported French bovine semen carrying BTV into China was scored according to the content of Supplementary Table S3 and calculated the total score after scoring according to the risk score description of influencing factors. As a result, the risk assessment score for the likelihood of the introduction of BTV through the bovine semen trade was $\text{P}{\text{A}}_{\text{The likelihood of bovine semen introduction}}=\sum_{\text{i}=1}^{\text{n}}{{\text{S}}_{\text{i}}\text{W}}_{\text{i}}=63.$ The full weighted score for the assessment of the likelihood of bovine semen trade being introduced into BTV was $\text{P}{\text{B}}_{\text{The likelihood of bovine semen introduction}}=\sum_{\text{i}=1}^{\text{n}}{{\text{S}}_{\max}\text{W}}_{\text{i}}=133$. Divide the obtained evaluation score by the full score value, and finally obtain the likelihood evaluation of bovine semen trade into BTV: ${\text{P}}_{\text{The likelihood of bovine semen introduction}}=\frac{\text{PA}{\;}_{\text{The likelihood of bovine semen introduction}}}{\text{PB}{\;}_{\text{The likelihood of bovine semen introduction}}}=\frac{\sum_{\text{i}=1}^{\text{n}}{\text{S}}_{\text{i}}{\text{W}}_{\text{i}}}{\sum_{\text{i}=1}^{\text{n}}{{\text{S}}_{\max}\text{W}}_{\text{i}}}=\frac{63}{133}=0.47$. Combined with the assessment of likelihood levels in risk events (Table 2), the likelihood level of risk event occurrence was medium.

#### Consequences levels of introducing BTV in bovine semen trade

According to Supplementary Table S4, the consequences of introducing Chinese imported bovine semen into BTV were scored, and the score was $\text{P}{\;}_{\text{The consequences of introducing bovine semen}}=\sum_{\text{i}=1}^{\text{n}}{{\text{S}}_{\text{s}}\text{W}}_{\text{s}}=42$ and the full score of weighted score is ${\text{PB}}_{\text{The consequences of introducing bovine semen}}=\sum_{\text{i}=1}^{\text{n}}{{\text{S}}_{\max}\text{W}}_{\text{s}}=90$. Divide the obtained evaluation score with the full score, and finally obtain the evaluation of the consequences of bovine semen trade into BTV: $\text{P}{\;}_{\text{The consequences of introducing bovine semen}}=\frac{\text{PA}{\;}_{\text{The consequences of introducing bovine semen}}}{\text{PB}{\;}_{\text{The consequences of introducing bovine semen}}}=\frac{\sum_{\text{i}=1}^{\text{n}}{\text{S}}_{\text{s}}{\text{W}}_{\text{s}}}{\sum_{\text{i}=1}^{\text{n}}{{\text{S}}_{\max}\text{W}}_{\text{s}}}=\frac{42}{90}=0.47.$ Combined with the assessment of consequence levels in risk events (Table 3), the consequence level of risk event occurrence was medium.

#### Risk levels of introducing BTV in bovine semen trade

**Figure 10:**
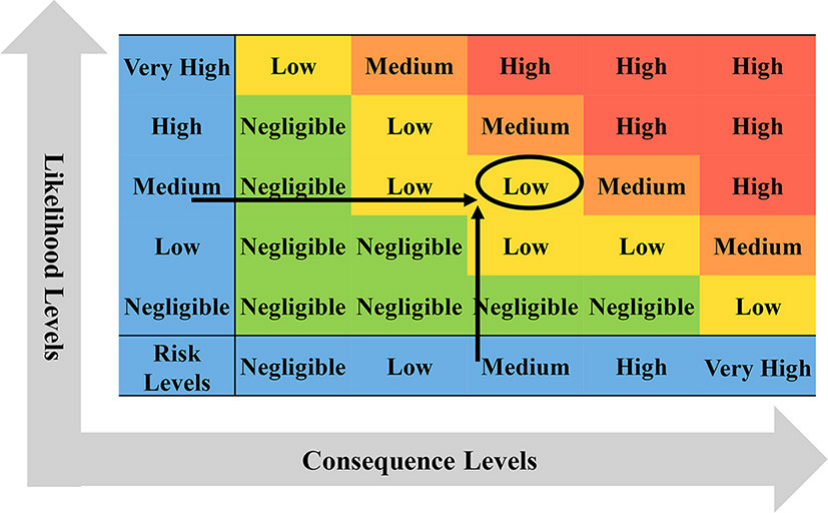
Risk determination matrix of BTV in France imported into China *via* bovine semen.

First, the likelihood of bovine semen carrying BTV into China was analyzed from the aspects of epidemic assessment, prevention and control measures, veterinary management system, and domestic quarantine. Then, the impact of BTV carrying bovine semen entering China on animal and human health and the domestic ecological environment, as well as the indirect impact on wider society and international trade, was evaluated. Finally, combined with the risk matrix in Figure 10, when the likelihood level of risk event occurrence was medium and the consequence level of risk event occurrence was medium, the risk level of BTV in France being introduced into China *via* bovine semen was low.

## Discussion

Bluetongue disease has appeared in Europe since 1956 (32). With the spread of different BTV strains in Europe, BT has not been effectively controlled in Europe (33). Especially from 2006 to 2008, the outbreak of BTV-8 in Europe brought huge economic losses to the European region (34). By 2008, the overall impact on the French finances was estimated to be 1.4 billion US dollars (35). The BTV-8 strain of the European epidemic period was virulent to both sheep and cattle. Genome sequence alignment showed that the BTV-8 outbreak in France in 2008 was similar to the BTV-8 strain isolated in sub-Saharan Africa (36). Therefore, some studies speculated that the BTV-8 in France may be derived from the attenuated vaccine in Africa (5). To cease the further spread of the disease in France, France launched a large-scale vaccination campaign and restrictions on trade in animals in March 2008. In 2012, France announces that BTV has been inactivated, but in September 2015, there was a recurrence of BT in central France (37). The clinical features of BT were observed in a sheep. Virus genome sequencing confirmed that the 2015 strain had 99.9% homology with the BTV strain popular in France in 2008, indicating the recurrence of the 2008 strain (38).

The recurrence of BT has led to a more severe outbreak of BT in France. According to the heatmap (Figure 5), the outbreak started at Louroux-de-Bouble in Auvergne, central France, and spread around. Due to insufficient doses of available inactivated vaccines in France in 2015, the French Veterinary Authority decided to implement control measures restricting the movement of livestock in order to limit the transmission of BTV from infected to non-infected areas and to establish a time period without *Culicoides* vector activity. However, BT has spread rapidly in mainland France as the disease has spread through the vector from the outbreak area to the unprotected area (2). The clinical symptoms of BT outbreaks are not obvious, which makes the diagnosis of BT difficult. There are reports showing that since 2015, BTV in France had mainly come from cattle tested before export; therefore, the number of BT in France may be seriously underestimated (5). In October 2016, sheep developed clinical signs of BTV in Corsica. Virus genome sequencing analysis revealed a close relationship between this strain and a strain isolated in Hungary in 2014 (39) and genetically distinct from a previous BTV strain circulating in Corsica. Therefore, it was suggested that the BTV in Corsica was not a recurrence of the previous strain but was introduced from other parts of Europe.

In the spatiotemporal scan analysis, when the maximum spatial scan window was set to 50% of the population at risk, the spatiotemporal aggregation area of BT in France cannot be well detected. On the one hand, when the spatial scanning window was 10 and 20%, the coverage was wide, and some surrounding countries and regions were covered, such as central Italy and Switzerland. On the other hand, when the population at risk increased, Corsica, a high outbreak area in France, has not been detected, which was prone to false negatives. Therefore, this study performed a comprehensive analysis using a scan ratio of 10–50% and found that the outbreak of BT in France from 2015 to 2018 was mainly concentrated in the south, and the time was concentrated from August to December. This result was consistent with the studies of Courtejoie (40) and Ward (41). BT gradually spread to southern France after the outbreak in 2007, and the infection of BTV was seasonal (in autumn and winter). In 2016, the number of BT outbreaks in France reached a peak, and the outbreaks were concentrated in mainland France. Afterward, BT continued to spread eastward in mainland France. By 2019, BTV had spread northeast to Switzerland, Germany, and Belgium. Research suggests that the spread of BT may be linked to the movement of infected livestock and *Culicoides* (42).

According to UN Comtrade Database, China's imports of live cattle are increasing. In 2020, China imported about 26,600 live cattle (27). France is the world's largest exporter of live cattle, and there is a potential trade possibility between China and France. Taking China as the research object, the risk of BTV through trade was analyzed. Infected live animals imported from disease-endemic countries may be potential carriers of BTV, especially if the animals are asymptomatically infected (43). BTV has an incubation period of ~10 days after initial infection when animals have no obvious clinical symptoms, and visual inspection of animals may fail to detect the disease, thus requiring laboratory testing in both exporting and importing countries. Since there is no record of live cattle trade between China and France in the past 10 years, two situations were assumed in the introduction of live cattle: China imports a small number of live cattle or imports a large number of live cattle. In the assessment of introducing BTV into China through trade, the risk of importing BTV from a large number of live cattle was higher than the risk of importing a small number of live cattle. In all trade risk analyses, the risk brought by a large number of imported live cattle was the highest. In the risk analysis of a large number of imported live cattle, since the French veterinary authorities listed BTV-8 and BTV-4 as endemic in France, it means that BTV will exist in France for a long time, and will continue to infect a large number of cattle and sheep every year (5), so the likelihood of introduction of live cattle carrying BTV into China was high (likelihood assessment was 0.51). At the same time, China's climatic condition is suitable for the survival of BTV and BTV transmitting Culicoides vector (10, 44), which may lead to the outbreak of BT in China (10, 44). In contrast, bovine semen, which is an animal product, is collected in semen collection centers with strict control measures and approved before entering. The impact after entering China is limited to breeding cattle, thus, the risk of the consequences was low. It is worth noting that the introduction of blight is a dynamic process, and the likelihood of introducing BT can change over time, especially with changes in global disease distribution or seasonal changes in vector transmission.

Risk matrices in the semi-quantitative analysis were used to identify risks, provide a quick estimate of risk, and are easy to use. The semi-quantitative analysis involves weighting and scoring each component and then identifying low, medium, and high risk. In this study, only trade invasion routes of live cattle and bovine semen were investigated. However, there may be more ways in which BT can invade (2). For example, the insect vector carrying the BTV enters the trading country through freighters and other means, so if these means are taken into account in the later stage, it is estimated that the risk of introduction of BT will be greater. At the same time, the risk of BTV is affected by the virulence of different strains, including the severity of the viral infection and the rapid control of disease transmission through vaccination. Therefore, with the further development of the model, the influencing factors of the model can be increased, decreased, or modified to adapt to the changing situation.

## Conclusion

This study conducted a spatiotemporal analysis of the BT epidemic in France, which showed that after the outbreak of BT in the central area of mainland France in 2015, there was a cluster outbreak of BT in France in 2016 and 2017, and then the epidemic shifted eastward to Switzerland, Germany, and Belgium. The semi-quantitative risk analysis results using China as an example showed that the risk of French BTV entering the trading country through live cattle trade is higher than the risk of BTV entering the trading country through bovine semen. The semi-quantitative risk analysis framework proposed in this study can explore possible invasive methods and influencing factors of BT. Therefore, it can provide rapid risk assessment for Sino-French animal trade in the future and a reference for other countries to quickly assess the risk of bluetongue transmission in import and export trade.

## Data availability statement

The raw data supporting the conclusions of this article will be made available by the authors, without undue reservation.

## Author contributions

BN and QC designed the study. Q-lY, S-wZ, S-yQ, and QZ collected the data. Q-lY, S-wZ, QZ, S-yQ, QC, and BN analyzed the data. All authors took part in interpreting the data and writing the manuscript. All authors contributed to the article and approved the submitted version.

## Funding

This study was supported by The National Key Research and Development Program of China (2016YFD0501101).

## Conflict of interest

The authors declare that the research was conducted in the absence of any commercial or financial relationships that could be construed as a potential conflict of interest.

## Publisher's note

All claims expressed in this article are solely those of the authors and do not necessarily represent those of their affiliated organizations, or those of the publisher, the editors and the reviewers. Any product that may be evaluated in this article, or claim that may be made by its manufacturer, is not guaranteed or endorsed by the publisher.
